# Palliative Surgical Approach to Rehabilitate Spinal Injury Patient in Indian Rural Setup

**DOI:** 10.4103/0973-1075.73646

**Published:** 2010

**Authors:** Pradeep K Singh, Harshal Sakale, Sandeep Shrivastva, Rajesh Dulani

**Affiliations:** Department of Orthopaedics and Trauma, Jawahar Lal Nehru Medical College & Acharya Vinoba Bhave Rural Hospital, Datta Meghe Institute of Medical Sciences, Wardha, India

**Keywords:** Conventional spinal instrumentation, Palliative care, Rehabilitation, Rural setup, Total traumatic paraplegia

## Abstract

**Objective:**

To evaluate the usefulness of conventional spinal surgery as palliative procedure to rehabilitate dorsolumbar injuries in a rural setup.

**Materials and Methods:**

Twenty-three patients with dorsolumbar spine injury with complete paraplegia were assessed on the clinical and social rehabilitation parameters after surgical stabilization at Acharya Vinoba Bhave Rural Hospital Sawangi, Wardha, India. The study group comprised 21 male and 2 female patients. The dorsolumbar spine injury was fixed by conventional posterior instrumentation using short-segment pedicle screw system and Harrington rod system with or without fusion. Functional and neurologic outcome was recorded in the follow-up period by using Functional Independence Measure and Frankel grade, respectively. Correlation and analysis of results was established statistically.

**Results:**

Functional outcome showed statistically significant improvement. Social cognition was found intact in a significant number of patients.

**Conclusion:**

This study demonstrates the usefulness of conventional instrumentation as palliative surgical approach to stabilize and rehabilitate patients from deprived sector of rural India.

## INTRODUCTION

Spinal injuries constitute one of the greatest calamities known to the medical world, causing great mortality and morbidity. Dorsolumbar spine trauma being associated with deformity and neurologic deficit is well known and this has been recorded since many years.[1–4] As with other traumatic injuries, in the past 3 decades, the shift in emphasis has been toward rapid mobilization and rehabilitation of the patient by various forms of internal fixation devices.[5–7] Newer advent in spinal stabilization system and rehabilitation science have allowed patients to regain mobility, improve their quality of life, and achieve prolonged survival after spinal cord injury.[8] The ultimate objective of treating spinal injury with deformity is to achieve spinal stability and rehabilitation. The authors feel that with the available health care infrastructure and financial constrains in rural India, conventional posterior instrumentation can be an option for most of the poor patients. The purpose of this study was to evaluate the usefulness of conventional instrumentation as palliative approach for traumatic dorsolumbar spine in the rural hospital setting.

## MATERIALS AND METHODS

In a retrospective study we included 25 patients with paraplegia after traumatic dorsolumbar spine between 2003 and 2009. Patients between 18 and 65 years of age with complete paraplegia (Frankel GradeA) after a dorsolumbar spine trauma were included in the study. Patients with head injury, polytrauma, and pathologic fractures were excluded. The fracture pattern was classified using McAfee classification system,[9] whereas Frankel grading system.[Box 1] was used to evaluate the neurologic status of the patients at different stages of follow-up.[10] The lesion was considered as spinal cord injury if the fracture was at D10 or above. If the fracture was between D11 and L2, it was considered a mixed conus medullaris and cauda equina injury. Anteroposterior and lateral radiographs were taken preoperatively to ascertain the status of the posterior elements, pedicles, and the amount of retropulsion of bony fragments into the spinal canal. In 9 patients, magnetic resonance imaging was performed whereas computed tomography scan was done in 3 patients. The patients underwent Harrington rod with or without sublaminar wiring (n = 13) or short-segment pedicle screw fixation (n = 12) along with decompressive laminectomy. Preoperative, postoperative, and follow-up films were measured in both planes to determine the angle of kyphosis and hardware condition. Surgeries were done by all the authors. The mean time for surgery was 155 min and the mean loss of blood was 250 mL. A thoracolumbar orthosis was prescribed for all the patients postoperatively, and we encouraged its use for approximately 8 weeks. Clinical, radiologic, and functional assessments were performed after 4 weeks followed by 8 weekly for 12 months. The functional assessment was done by an independent occupational therapist by using Functional Independence Measure (FIM) grading [Box 2] whereas neurology was assessed clinically and documented using the Frankel grading.

**Box 1**

Frankel scale[9]

- Absent motor and sensory function
- Sensation present, absent motor function
- Sensation present, motor function present but not useful (Grade 2–3/5)
- Sensation present, motor function present and useful (Grade 4/5)
- Normal motor and sensory function.

**Box 2**

Functional Independence Measure

**SELF-CARE**

- Grooming
- Bathing
- Dressing – Upper
- Dressing – Lower
- Toileting

**SPHINCTER CONTROL**

F. Bladder

G. Bowel

**TRANSFERS**

H. Bed, chair, wheelchair

I. Toilet

J. Tub shower

**LOCOMOTION**

K. Walker/wheelchair

L. Expression

**COMMUNICATION**

M. Comprehension

N. Expression

**SOCIAL COGNITION**

O. Social interaction

P. Problem solving

Q. Memory

FIM, Functional Independence Measure.

**FIM Level**

**No Helper**

7. Complete independence (timely; safety)

6. Modified independence (device)

**Helper – Modified dependence**

5. Supervision (Subject = 100% or more)

4. Minimal assistance (subject75% or more)

3. Moderate assistance (subject 50% or more)

**Helper – Complete dependence**

2. Maximal assistance (subject 25% or more)

1. Total assistance or not testable (subject less than 25%)

### Statistics

Student paired *t* test was used to assess the statistical difference in the functional outcome and deformity correction.

## RESULTS

**Figure 1:**
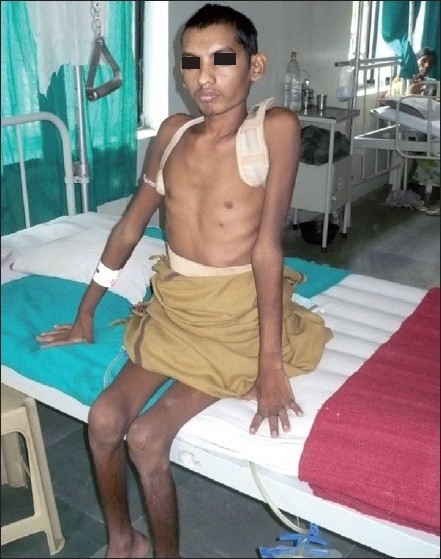
A clinical photograph of a 22-year-old male patient with complete paraplegia being rehabilitated in the ward on the 7th postoperative day

**Figure 2:**
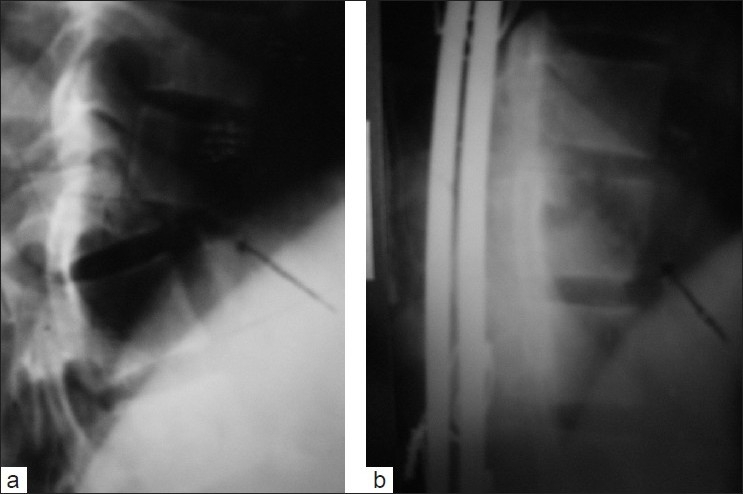
(a) A lateral radiograph of dorsolumbar spine of a 22-year-old male patient shows fracture dislocation of D11 with severe kyphotic deformity along with fracture of superior end plate of D11 vertebral body (arrowhead), presented with complete paraplegia. (b) Harrington rod and sublaminar wiring fixation done extending 2-level cephalad and 3-level caudalad demonstrating excellent correction of kyphosis and restoration of the vertebral height

**Figure 3:**
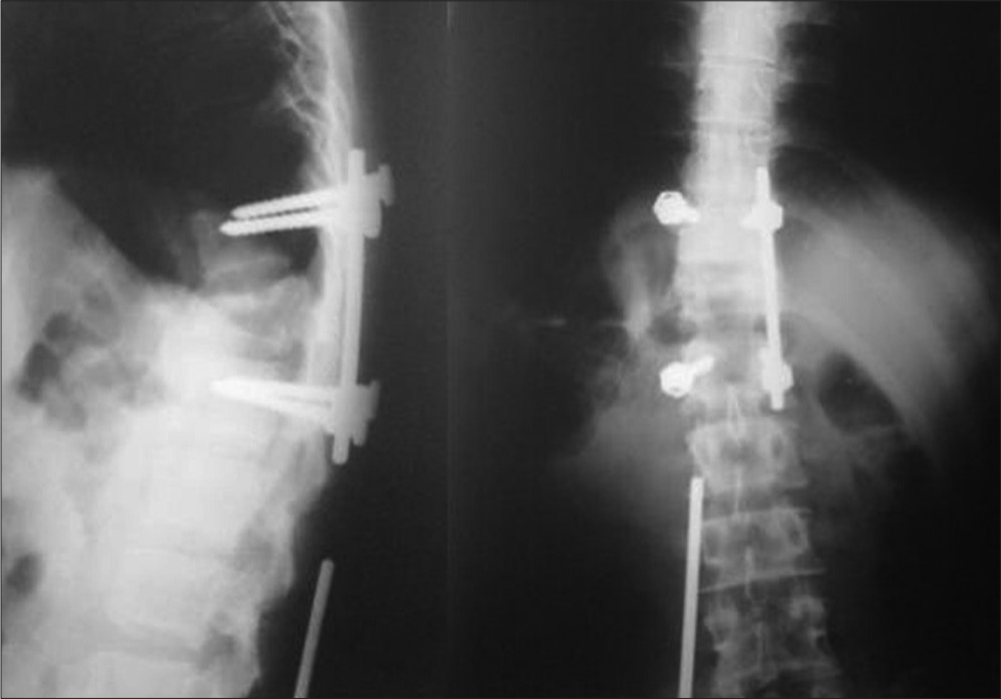
Radiograph revealing loosening of implant and migration of connecting rod

**Chart 1:**
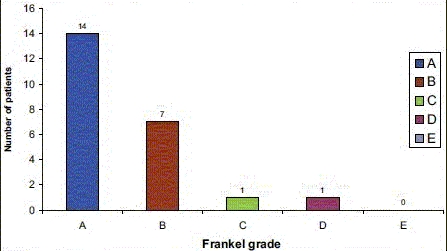
Table demonstrates neurological status of the patients at the end of the follow-up

A majority of the patients had no financial or emotional backup. One patient lost to follow-up and 1 patient died due to general debility. The remaining 23 patients completed the study. There were 21 male and 2 female. The mean age was 30.8 years. Fall from height while working for the daily wages was the most common (70%) cause of injury. Trauma at dorsal lumbar junction formed the bulk of the injured vertebrae (60.8%). Wedge compression fracture was the most common fracture type observed in 47.8% [Table 1] of patients. All the patients were having complete paraplegia (Frankel Grade A) at the time of presentation. The patients were brought to the hospital within 2–40 days (mean 7 days). The patients were followed up to mean period of 12.4 months. The results were encouraging when observed for their social life. Most of the patients were leading psychologically adjusted lives and showed a significant improvement in the FIM score from 48.50 to 79.33 [Table 2]. Sixteen patients scored high in self-care as well as in social cognition. Eighteen patients were able to achieve ambulatory status using crutches, walker, or a wheelchair at the end of the follow-up [Figure 1]. The mean score of kyphotic deformity was 16.5° in preoperative radiograph, whereas postoperative radiograph showed a mean of 5.4° [Figure 2a and b] at the end of the follow-up. Ten patients demonstrated poor scores in bladder and bowel care despite bladder rehabilitation training. At 6 months, all the patients demonstrated a fair evidence of fracture union and 17 patients maintained hardware position in situ with significant correction of deformity. Only 1 patient demonstrated major loosening of implant [Figure 3]. Four patients had superficial infection. Bed sore was the concern for 6 patients of whom 5 sores healed with dressing and debridement subsequently. Urinary tract infection was present in 5 patients. One patient developed refractory infection of the urinary tract. Urinary tract infection and bedsore both were present in 1 patient even after completion of the study. This was the only patient who showed poor outcome in all the parameters. The mean neurologic recovery was 0.5 Frankel grade. Two patients recovered up to Grade C and D on Frankel scale, 7 patients improved neurologically by Grade 1 and reached up to Grade B, whereas 14 patients did not show any improvement in neurology [Chart 1]. Statistical analysis revealed insignificant difference in the outcome (*P* value 0.68).

**Table 1:** Type of fracture McAfee’s classification

Type of fracture	Number of patients	Percentage
Wedge compression	11	47.8
Stable burst fracture	3	13.0
Unstable burst fracture	2	8.6
Fracture dislocation	5	26.1
Translational injury	2	8.6

**Table 2:** FIM score and deformity

	FIM score (mean)	Kyphotic deformity (mean)
Preoperative	48.50	16.6°
At the end of follow-up	79.33	5.4°
*P* value*	<0.001	<0.001

FIM, Functional Independence Measure; Table demonstrates improvement in both FIM score and angle of kyphosis in the pretreatment and on last follow up with significance;

* *P* value < 0.001 single-tailed Student paired *t* test

## DISCUSSION

A body of information now supports the view that early spinal fixation and mobilization in traumatic spinal injuries cause potential reduction in the psychologic problems of prolonged recumbency. There is also evidence that the incidence of medical complications is lower, or at least no greater, with early surgical treatment.[2,3] FIM was used to demonstrate final functional and psychologic state of the patients; 82.6% of our patients were able to achieve ambulatory status using a wheel chair, a caliper, or a walker by 12 months and were earning their livelihood. This again substantiates the need for early stabilization of spine to quickly mobilize the patient with an aim of early functional rehabilitation.[11] Psychologic adjustment of our patient is attributed to denial, which is a very strong and appropriate mechanism for dealing with a devastating loss.[11,12] The level of injury does not always reflect the severity of the emotional response as we found similar observation in our study. Management of bladder problems in spinal cord injury patients is an integral aspect of treatment.[13] We believe that the illiteracy and lack of family support in a majority of the rural Indian population prevents them from understanding the importance of asepsis., although we tried to train them for self-intermittent catheterization as long as they were indoor.

Conventional Harrington rod and posterior short-segment stabilization have their own biomechanical limitations in unstable injuries.[14] Considering the low socioeconomic strata of our patients, we used conventional instrumentation irrespective of the type of spine injury. There are studies in the literature on patients with complete traumatic paraplegia suggesting improvement in neurologic level by Grade 2 Frankel scale after stabilization of the spine.[15] In our study, the patients showed an average improvement of 0.5 Grade on Frankel grading, which is not satisfactory after comparing with the literature.[15] Delayed presentation, transportation of patients to the hospital, and delay in surgery could be the reasons to explain this.

In conclusion, this study demonstrates the usefulness of conventional posterior instrumentation as palliative surgery and rehabilitation tool, in traumatic dorsolumbar spine in rural institute where financial and infrastructure constrains limit the outcome.
